# Inflammation-induced miRNA-155 inhibits self-renewal of neural stem cells via suppression of CCAAT/enhancer binding protein β (C/EBPβ) expression

**DOI:** 10.1038/srep43604

**Published:** 2017-02-27

**Authors:** Kayoko Obora, Yuta Onodera, Toshiyuki Takehara, John Frampton, Joe Hasei, Toshifumi Ozaki, Takeshi Teramura, Kanji Fukuda

**Affiliations:** 1 Department of Rehabilitation Medicine, Kindai University Faculty of Medicine, Osaka, Japan; 2 Division of Cell Biology for Regenerative Medicine, Institute of Advanced Clinical Medicine, Kindai University Faculty of Medicine, Osaka, Japan; 3 School of Biomedical Engineering, Dalhousie University. Halifax, Nova Scotia, Canada; 4 Science of Functional Recovery and Reconstruction, Okayama University Graduate School of Medicine, Dentistry and Pharmaceutical Sciences, Okayama, Japan

## Abstract

Intracerebral inflammation resulting from injury or disease is implicated in disruption of neural regeneration and may lead to irreversible neuronal dysfunction. Analysis of inflammation-related microRNA profiles in various tissues, including the brain, has identified miR-155 among the most prominent miRNAs linked to inflammation. Here, we hypothesize that miR-155 mediates inflammation-induced suppression of neural stem cell (NSC) self-renewal. Using primary mouse NSCs and human NSCs derived from induced pluripotent stem (iPS) cells, we demonstrate that three important genes involved in NSC self-renewal (*Msi1, Hes1* and *Bmi1*) are suppressed by miR-155. We also demonstrate that suppression of self-renewal genes is mediated by the common transcription factor C/EBPβ, which is a direct target of miR-155. Our study describes an axis linking inflammation and miR-155 to expression of genes related to NSC self-renewal, suggesting that regulation of miR-155 may hold potential as a novel therapeutic strategy for treating neuroinflammatory diseases.

Inflammation is linked to increased neuronal degeneration, neural cell death, altered gene expression profiles and changes in cell physiology in a variety of intractable neurological and cerebrovascular diseases^1^,^2^. Severe chronic neuronal inflammation can lead to irreversible neuronal damage and loss of cell populations responsible for essential sensory, motor and cognitive functions. Neural cells in the hippocampus, especially neural stem cells (NSCs), are vulnerable to inflammatory stress^3^. Exposure to inflammatory signals suppresses self-renewal of NSCs in the adult brain^4^,^5^,^6^. This loss of neurogenesis is restored by blocking inflammation^4^,^5^, suggesting that there may be an intricate molecular network linking neural inflammation to stem cell renewal in the brain. Elucidating this network may support development of therapeutics for treating diseases associated with neuronal inflammation. Integrity of neural stem cells (NSCs) is maintained by transcription factors, epigenetic modifiers and non-coding RNA (ncRNA)^7^,^8^,^9^. Sox2, Hes1, Nestin, Bmi1 and Musashi-1have been used as markers for NSCs^10^. Among these, expression of Hes1 (a bHLH transcription factor involved in stem cell maintenance)^11^,^12^, Bmi-1 (a polycomb group RING finger protein required for NSC maintenance)^13^,^14^ and Musashi-1 (Msi-1; an RNA-binding protein highly expressed in proliferating NSCs)^15^, are down-regulated through neuronal differentiation and are used as markers for undifferentiated NSCs. In contrast, expression of Prox1, NeuroD1, doublecortin (DCX), GFAP, Nestin and Neurofilament-M (Nf-M)^16^ are expressed in both progenitor cells and committed cells^17^.

Recently, miRNAs have emerged as important regulators of a wide range of biological processes, including diseases associated with inflammation^18^,^19^,^20^,^21^,^22^,^23^. This class of small 21–23-nucleotide-long single-stranded RNAs bind to the 3′ UTR regions of target mRNAs, resulting in gene silencing either by interfering with protein translation or by degradation/cleavage of the mRNA transcript^22^,^24^,^25^,^26^. Among the many miRNAs that have been identified, miR-155 is highly conserved across vertebrate species and is thought to be among the most important miRNAs involved in inflammatory responses^19^,^27^. Pro-inflammatory signals such as interleukin (IL)-1, IL-6 and tumor necrosis factor (TNF)-α upregulate miR-155 by way of transcriptional activation. In addition, miR-155 suppresses expression of anti-inflammatory molecules, such as SOCS-1 and SOCS-3^28^,^29^,^30^,^31^. Recently, it has been discovered that miR-155 is increased in inflammatory CNS disorders such as amyotrophic lateral sclerosis (ALS)^32^, multiple sclerosis (MS)^33^ and Down’s syndrome^34^. Furthermore, inhibiting miR-155 with complementary anti-miRNA oligonucleotides reduces impairment in animal models of MS and ALS^32^,^35^.

Collectively, these studies suggest that miR-155 represents a promising target for anti-inflammatory therapy in the CNS. However, the detailed molecular mechanisms linking miR-155-mediated inflammation with suppression of genes related to NSC self-renewal and maintenance remain unclear. In this study, we examine the relationship between miR-155 and stem cell self-renewal in primary mouse NSCs and human NSCs derived from induced pluripotent stem (iPS) cells.

## Materials and Methods

### Antibodies and reagents

The following commercially-available antibodies were used: rabbit anti-GAPDH polyclonal antibody (sc-25778, Santa Cruz Biotechnology, CA, USA. 1:5,000 dilution), mouse anti-Musashi-1 monoclonal antibody (14H1, 14-9896, eBioscience, CA, USA. 1:1,000 dilution), rabbit anti-Hes1 polyclonal antibody (GTX108356, Gene Tex, CA, USA. 1:2,000 dilution), rabbit anti-Bmi1 polyclonal antibody (5856, Cell Signaling Technology, MA, USA. 1:2,000 dilution), mouse anti-Nestin monoclonal antibody (Rat-401, 14-5843, eBioscience. 1:1,000 dilution), mouse anti-Nf-M monoclonal antibody (RMO 14.9, 2838, Cell Signaling Technology. 1:1,000 dilution), rabbit anti-C/EBPα XP polyclonal antibody (D56F10, 8178, Cell Signaling Technology. 1:2,000 dilution), rabbit anti-C/EBPβ polyclonal antibody (LAP, 3087, Cell Signaling Technology. 1:2,000 dilution), and rabbit anti-Caspase-3 polyclonal antibody (9662, Cell Signaling Technology. 1:3,000 dilution). Secondary antibodies were goat anti-mouse IgG HRP (sc-2005, Santa Cruz Biotechnology. 1:50,000 dilution) and goat anti-rabbit IgG HRP (sc-2004, Santa Cruz Biotechnology. 1:50,000 dilution). Recombinant human IL-1β (211-11B, PeproTech, NJ, USA) was prepared according to the manufacturer’s instructions.

### Establishment and culture of NSCs from mice pups

All procedures involving animals were approved by the Institutional Animal Care and Use Committee at Kindai University, and were performed in accordance with institutional guidelines and regulations. The B6D2F1 mice used in this study were obtained by crossing C57BL/6 N female mice with DBA/2 J male mice. NSCs were prepared from 2-day-old B6D2F1 pups. Whole brains collected from B6D2F1 pups following euthanasia and dissection were homogenized by scalpel. The homogenized brains were mixed with fresh DMEM medium and filtered using a 40 μm cell strainer (BD Falcon^TM^, NJ, USA). Single cell suspensions containing stem cells were plated onto Petri dishes (BD Falcon^TM^) and cultured in NSC medium consisting of StemSure^®^ DMEM (Wako, Tokyo, Japan), StemSure^®^ Serum Replacement (SSR, 197-16775, Wako), MEM Non-essential amino acids, 0.1 mM 2-mercaptoethanol (Thermo Fisher Scientific, CA, USA), 200 mM L-glutamine (Thermo Fisher Scientific), 10 ng/ml Fibroblast growth factor 2 (RCHE0T003, Reprocell, Tokyo, Japan) and 20 ng/ml Epidermal growth factor (E9644, SIGMA-Aldrich, Inc., MO, USA.). On day 2 of culture, the medium was replaced to remove dead cells and debris. After 7 days of culture, NSCs that aggregated into clumps with smooth surfaces (i.e., neurospheres) were selected and disaggregated by treatment with Accutase (AT104-500, Innovative Cell Technologies, Inc., CA, USA.) and passaged in the fresh NSC medium. NSCs were passaged no more than two times for use in subsequent experiments.

### Cell counting

We used 4 NSC culture plates per experiment, which were prepared with the same number of cells and maintained under identical culture conditions. For cell counting, one of these plates was randomly selected at 24 hrs, 48 hrs and 72 hrs of culture, transferred to a 15 ml conical tube (BD Falcon), centrifuged and then treated with TrypLE for 5 minutes after removing the supernatant. Dissociated cells were stained with trypan blue and counted using a TC20 automated cell counter (Bio-Rad Laboratories, CA, USA).

### Real-time RT-PCR for miRNA

Total RNA was collected from the NSCs in each experiment using TRI Reagent^®^ (TR118, Molecular Research Center, Inc., OH, USA) and the PrimeScript^®^ RT Master Mix Kit (RR036A, TAKARA Bio Inc., Shiga, Japan). Quantitative real-time PCR of total cDNA was performed using Perfect real-time SYBR green II (RR820L, Takara). PCR amplifications were performed on a Thermal Cycler Dice^®^ Real Time System Single at 95 °C for 20 s followed by 40 cycles at 95 °C for 5 s and 60 °C for 30 s. To quantify the relative expression of each gene, the Ct (threshold cycle) values were normalized by GAPDH (ΔCt = Ct_target_ − Ct_GAPDH_) and compared with a calibrator using the “ΔΔCt method” (ΔΔCt = ΔCt_sample_ − ΔCt_control_). Data were expressed as mean values ± SD of 3 experiments. Statistical significance was evaluated by Student’s t-test with JMP software version 10.0.0 (SAS Institute, Cary, NC, USA). Primer sequences are listed in Supplementary Table S1.

### Real-time RT-PCR analysis for mature miR-155

For miRNA qPCR, total RNA prepared as above was reverse-transcribed using the Universal cDNA synthesis Kit II (Exiqon, Inc., Vedbaek, Denmark). The resulting cDNA was diluted 1:50 for qPCR. The PCR was performed using an ExiLENT SYBR^®^ Green master mix (Exiqon) with the following miRCURY LNA PCR primer sets (Exiqon):mmu-miR-15-5p (ID 205930) and U6 snRNA (ID 203907). To obtain the relative expression, the Ct (threshold cycle) values of miR-155 were normalized by expression of U6 (ΔCt = Ct_miR−155_ − Ct_U6_) and compared with a calibrator using the “ΔΔCt method” (ΔΔCt = ΔCt_sample_ − ΔCt_control_). Data were expressed as mean values ± SD of 3 experiments. Statistical significance was evaluated by Student’s t-test with JMP software version 10.0.0 (SAS Institute, Cary, NC, USA).

### Western blot (WB) based analysis

The NSCs treated in each experiment were homogenized in SDS buffer (4% SDS, 125 mM Tris–glycine, 10% 2-mercaptoethanol, 2% bromophenol blue in 30% glycerol) and centrifuged at 9,000 g for 10 min at 4 °C to remove debris. Aliquots were subjected to polyacrylamide gel electrophoresis in the presence of SDS (SDS/PAGE) followed by electrotransfer onto a PVDF membrane (Hybond-P; GE Healthcare, Tokyo, Japan). The blotted membranes were blocked overnight with Block Ace (UKB80, Dainippon Sumitomo Pharma) and then probed with primary antibodies overnight at 4 °C. Detection was performed with horseradish peroxidase (HRP)-conjugated secondary antibodies (all from Santa Cruz Biotechnology) and either ECL prime (GE Healthcare) or Immunostar ^®^ LD (290-69904, Wako) detection reagents. The lumino-labeled membranes were analyzed on an Amersham™ Imager 600 CCD-based chemiluminescent analyzer (GE Healthcare). For assessment of protein expression levels, relative band intensities were quantified using ImageQuant™ TL software (GE Healthcare). All data were reported as mean values ± SD of 3 experiments.

### Transfection of siRNA, miRNA inhibitor and plasmids

Prior to transfection, NSCs were dissociated using TrypLE Express, washed twice with PBS and placed in 100 μl of Opti-MEM (31985-070, Thermo Fisher Scientific). The NSCs were then transfected with miR-plasmids, siRNAs, miRNA inhibitors or pPBQM-miR-155 plasmid using a CUY21 electroporator (NEPA Gene, Tokyo, Japan). For over-expression experiments, we used the miExpress™ Precursor miRNA Expression system (GeneCopoeia Inc. MD, USA). In this system, the precursor miRNA is placed in the 3′ region of the EGFP gene. After transfection, the precursors are co-expressed with EGFP and processed in the cells. We used a scrambled control sequence expression plasmid (CmiR0001-MR04, GeneCopoeia, Inc.) and an mmu-miR-155-5p precursor expression plasmid (MmiR3427-MR04, GeneCopoeia, Inc.). To observe the effect of miRNA on NSC proliferation, we used the pPBQM-miR-155 plasmid (a gift from Dr. Martin Lotz of The Scripps Research Institute, CA, USA), which consists of the precursor sequence of mmu-miR-155 controlled by a cumate-gene switch, an EF1-CymR repressor cassette and a piggyback transposon backbone. In this system, the induction of miR-155 transcription relies on the addition of cumate, which is a non-toxic water-soluble small molecule^36^. The cumate-gene switch was activated by adding 30 ug/ml cumate (QM100A-1, System Bioscience, Inc. CA, USA).

For miRNA inhibition experiment, we used an miRNA inhibitor for miR-155 (*mir*Vana^®^ miRNA inhibitor to mmu-miR-155, MH13058 and to hsa-miR-155, MH12601, both from Thermo Fisher Scientific) with Lipofectamine^®^ RNAiMAX (Thermo Fisher Scientific) following the manufacturer’s instructions. For siRNA mediated C/EBPβ suppression, we used Silencer^®^ Select siRNA (s127565, Thermo Fisher Scientific) with a scrambled control (Silencer Select Negative Control, Thermo Fisher Scientific). To observe the effects of high-doses of miR-155 in the human cells, we used miRNA mimic (MC12601, Thermo Fisher Scientific).

### Chromatin Immunoprecipitation (ChIP) assays

ChIP assays was performed using a ChIP-IT Express Magnetic Chromatin Immunoprecipitation kit (53009, Active motif, CA, USA) following the manufacturer’s instructions. Briefly, the cells were fixed in formaldehyde and the chromatin was enzymatically digested. The chromatin was then immunoprecipitated at 4 °C overnight using 2 μg anti-C/ebpβ antibody (sc-150x, Santa Cruz Biotechnology) and Normal mouse IgG antibodies (Santa Cruz Biotechnology). The immunoprecipitates were collected using magnetic protein G beads and then washed three times. After the chromatin was eluted from the beads, cross-links were removed by treatment with reverse cross-linking buffer. The immunoprecipitated DNA was used to detect Msi1, Hes1 and Bmi1 promoter sequences by standard PCR. The following ChIP primers were used: Msi1 sense 5′-ATGTGGCTCAGCAGGTTGAG-3′; Msi1 antisense 5′-TGCTGCTGTTCACCTTGATGC-3′; Hes1 sense 5′-TGTCTTGGCCTATATCTGTTC-3′; Hes1 antisense 5′-ACTCTTTCCTCTGGGCTTTGC-3′, Bmi1 sense 5′-ACTACACCGACACTAATTCCCAG-3′ and Bmi1 antisense 5′-TCCAAAATGGCTCGGAGTCC-3′. Input DNA represented 5% of the total chromatin used in each reaction before immunoprecipitation.

### Differentiation of human iPS cells

The 409B2 human iPS cell line^37^ was cultured in complete Essential 8 medium (A1517001, Thermo Fisher Scientific) on culture dishes coated with iMatrix-511^™^ (892011, Wako). For NSC-induction, iPS colonies were dispersed to single cells with TrypLE Express^™^ (12605-028, Thermo Fisher Scientific) and then centrifuged. Cells were re-suspended in PSC-Neural induction medium^™^ (1647801, Thermo Fisher Scientific) and cultured on Matrigel^™^(354234, Corning, NY, USA)-coated dishes for 1 week. NSC differentiation was confirmed by differentiation to NESTIN-positive and NF-M positive neural cells.

### Immunofluorescence

Differentiated NSCs were washed, fixed with 4% paraformaldehyde for 30 min, washed three times with 0.2% Triton-X100 in PBS and blocked with Block Ace. The cells were then reacted with anti-Nestin or anti-Nf-M primary antibodies overnight at 4°C. Subsequently, the cells were washed three times with 10%Block Ace/PBS and incubated with FITC-conjugated goat anti-mouse IgG secondary antibody (Santa Cruz Biotechnology) in 10%Block Ace/PBS for 1 h at room temperature. The cells were washed again with 0.2% Triton-X100/PBS and then stained with DAPI-containing PBS. Fluorescence was detected using a BZ-X710 All-in-One Fluorescence Microscope (KEYENCE, Osaka, Japan).

### Statistical analysis

Significant differences were detected by Tukey-Kramer HSD test or Student’s t-test, as appropriate. P values less than 0.05 were considered significant.

## Results

### IL-1β attenuates stem cell-related gene expression in mouse NSCs

**Figure 1:**
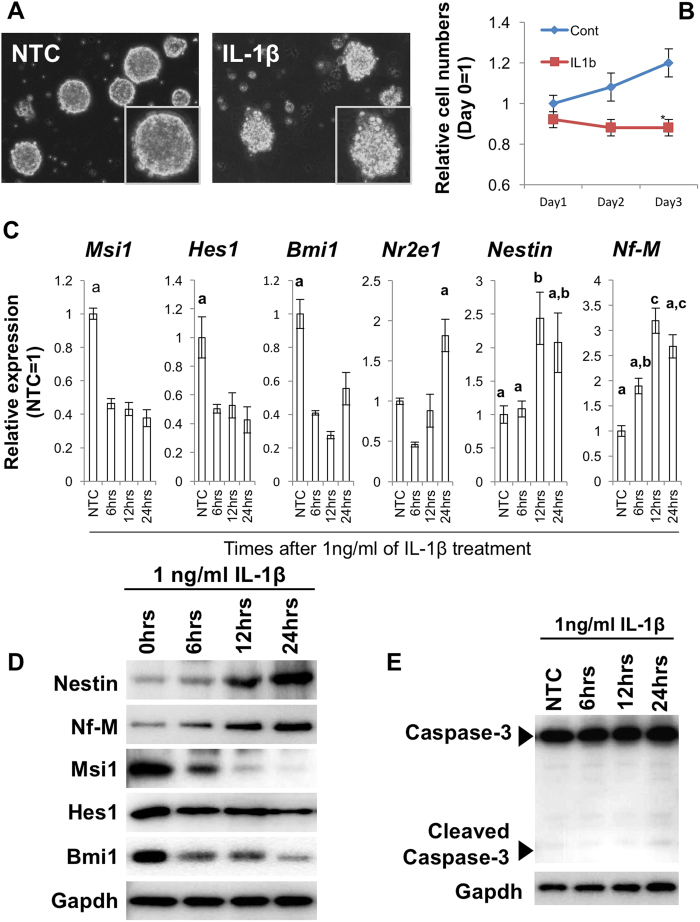
IL-1β suppresses stem cell self-renewal gene expression in mouse NSCs. (**A**) Morphological changes of NSCs treated with 1 ng/ml IL-1β. (**B**) Difference in cell proliferation between mock control (PBS, Cont) and 1 ng/ml IL-1β-treated cells. Cell numbers were normalized by initial cell number (1 × 10^5^ cells/ml). The asterisks represent a significant difference (P < 0.05) between the groups. (**C**) qPCR for genes related to NSC self-renewal. Specimens were collected at each time point after 1 ng/ml IL-1β administration. Characters a–c represent significant differences among groups (P < 0.05) determined by Tukey-Kramer HSD test for multiple comparison. Briefly, the different characters above each column (*e.g.* a and b) represent significant differences among the columns and the same characters above different columns (*e.g.* a and a) show no significant difference. (**D**) Western blots for Nestin, Nf-M, Msi1, Hes1 and Bmi1 for NSCs treated with 1 ng/ml IL-1β administration. (**E**), Western blot for Caspase 3, demonstrating no measurable change cleaved-caspase related to apoptosis.

We examined the effects of exposure to the inflammatory cytokine IL-1β on mouse NSC self-renewal. When exposed to 1ng/ml IL-1β, neurospheres displayed abnormal morphologies characterized by the appearance of weakly attached cells at the peripheries (Fig. 1A). NSCs from these neurospheres did not proliferate over the course of 3 days of IL-1β treatment (Fig. 1B). Relative expression of the NSC marker genes *Msi1, Hes1* and *Bmi1* was also markedly lower after 6, 12 and 24 hours of IL-1β treatment. On the other hand, the relative expression of *Nr2e1, Nestin* and *Nf-M*, which are markers for committed cells, significantly increased (Fig. 1C and D). To eliminate the possibility that alterations in relative gene expression resulted from cell death, we examined Caspase-3 expression. The activated (cleaved) form of Caspase-3 was not detected by Western blot (WB) (Fig. 1D). Based on these initial experiments, we hypothesized that IL-1β inhibits NSC self-renewal by modifying gene expression.

### MiR-155 is involved in IL-1β-induced suppression of self-renewal genes

**Figure 2:**
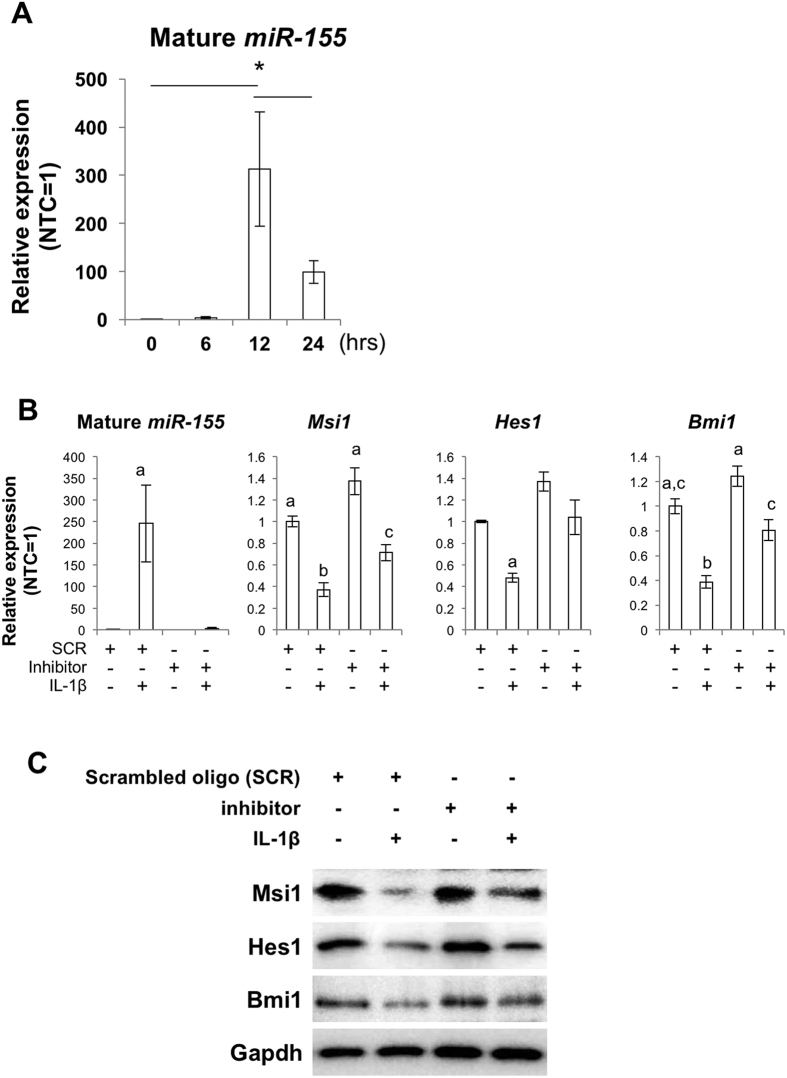
miR-155 is involved in IL-1β-induced suppression of self-renewal genes. (**A**) IL-1β-induced expression of mmu-miR-155 (miR-155). The y-axis represents expression relative to the no-treatment control (NTC). U6 small nuclear RNA (snRNA) was used as an internal control. The asterisks represent a significant difference (P < 0.05) between the groups. (**B**) qPCR for mature *miR-155, Msi1, Hes1* and *Bmi1* in NSCs treated with the scrambled oligonucleotide (SCR, control), the miRNA inhibitor oligonucleotide to mmu-miR-155 (inhibitor) and 1 ng/ml IL-1β. Treatment with the inhibitor ameliorated suppression of *Msi1, Hes1* and *Bmi1* expression by IL-1β. Characters a-c represent significant differences among groups (P < 0.05) determined by Tukey-Kramer HSD test for multiple comparison. (**C**) Western blots for Msi1, Hes1 and Bmi1 for NSCs treated with the SCR, inhibitor and IL-1β.

To examine the possibility that miR-155 mediates the IL-1β-induced suppression of stem cell self-renewal, we measured expression levels of miR-155 in NSCs. Using miR-qPCR to detect the mature form of the target miRNA, we observed a significant increase in expression of miR-155 after 12 and 24 hours of 1 ng/ml IL-1β treatment (Fig. 2A). To determine if inhibition of miR-155 could ameliorate the IL-1β effect on NSCs, we pretreated the NSCs with an miR-inhibitor to mmu-miR-155-5p 24 hours before IL-1β stimulation. When miR-inhibitor pre-treated NSCs were exposed to IL-1β for 12 hours, levels of NSC marker genes remained close to baseline levels observed for control cells treated with the scrambled oligonucleotide (SCR) (Fig. 2B and C).

### Over-expression of miR-155 disrupts NSC self-renewal

**Figure 3:**
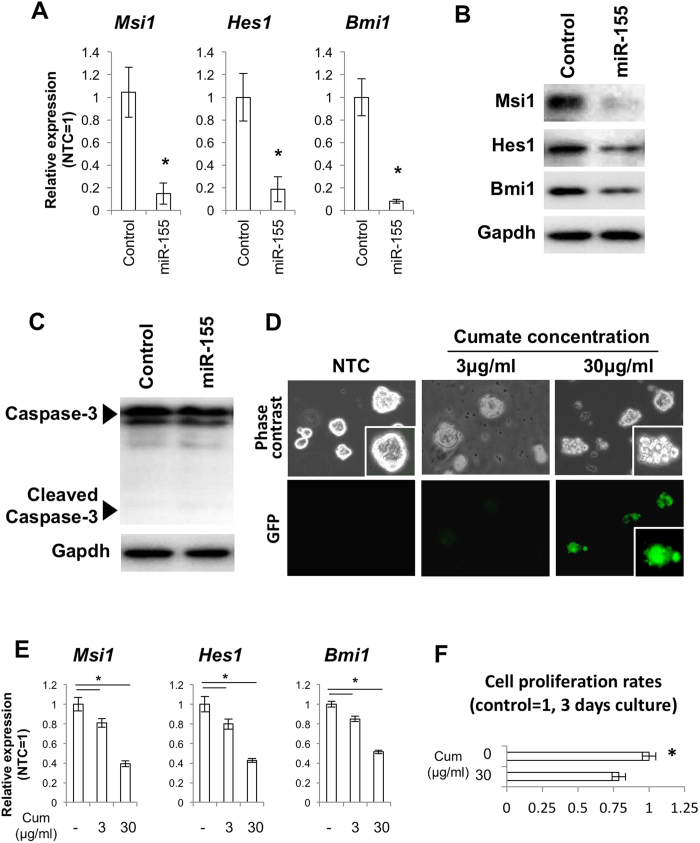
Over-expression of miR-155 leads to suppression of the self-renewal genes *Msi1, Hes1* and *Bmi1* and inhibition of self-renewal. (**A**) qPCR for *Msi1, Hes1* and *Bmi1* for NSCs transfected with the GFP-NTC (control) and the GFP-mmu-miR-155 (miR-155) plasmids. Asterisks represent significant differences (P < 0.05) compared with control. (**B**) Western blots for Msi1, Hes1 and Bmi1 for NSCs transfected with the control and miR-155 plasmids. (**C**) Western blot for caspase 3 for NSCs transfected with control and miR-155 plasmids. (**D**) Cumate induction of miR-155 and GFP expression in NSCs transfected with pPBQM-miR155-IRES-GFP. (**E**) qPCR for *Msi1, Hes1* and *Bmi1* for NSCs stably expressing pPBQM-miR155-IRES-GFP (QM-miR155). Asterisks represent significant differences (P < 0.05) among groups. (**F**) Rates of cell proliferation for NSCs stably expressing QM-miR155 with and without cumate treatment. Cell numbers were normalized by cell numbers for the control group at 3 days of culture. The asterisk represents a significant difference (P < 0.05).

To directly examine the effect of miR-155 on expression of *Msi1, Hes1* and *Bmi1*, we over-expressed miR-155 using a plasmid encoding the pri-miR-155 cDNA sequence in the 3′UTR of the GFP gene. Expression of *Msi1, Hes1* and *Bmi1* decreased by approximately 80% compared to control NSCs, in which GFP with a scrambled sequence was expressed (Fig. 3A). Suppression of Msi1, Nestin and Bmi1 was also confirmed at the protein level (Fig. 3B). A WB for Caspase-3 indicated that over-expression of miR-155 did not affect NSC viability (Fig. 3C). To independently confirm the effect of miR-155 over-expression on NSC self-renewal, we produced NSCs possessing a cumate inducible miR-155 system and observed target gene expression after cumate supplementation. Induction of miR-155 was monitored by presence of GFP co-expressed by IRES sequence (Fig. 3D). Induction of miR-155 by cumate resulted in suppression of *Msi1, Hes1* and *Bmi1* (Fig. 3E), accompanied by morphological changes in the neurospheres and inhibition of cell proliferation (Fig. 3D and F).

### MiR-155 attenuates NSC-related gene expression through suppression of C/ebpβ

**Figure 4:**
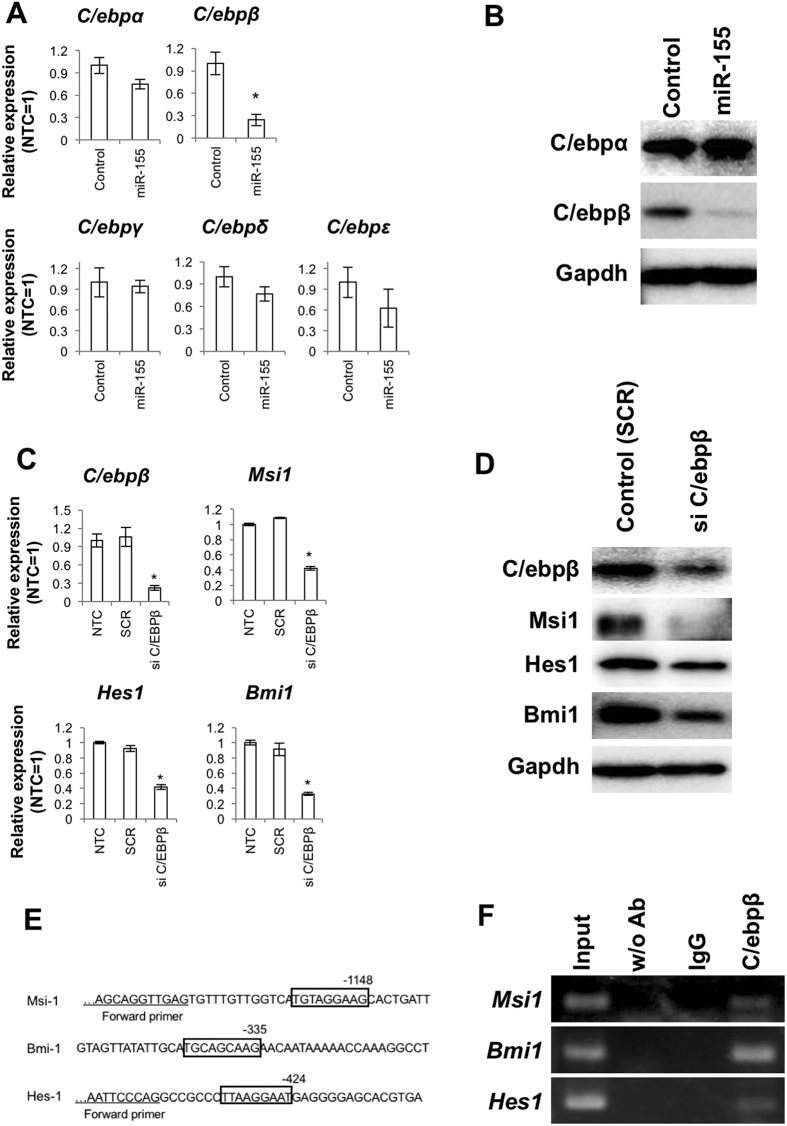
C/EBPβ is involved in miR-155-mediated attenuation of the stem cell self-renewal genes in NSCs. (**A**) Quantitative RT-PCR for *C/EBP* family members in NSCs overexpressing miR-155. The control was NSCs transfected with a scrambled sequence. The asterisk represents a significant difference (P < 0.05) compared with the control. (**B**) Western blots for C/EBPα and β after overexpression of miR-155. The control was NSCs transfected with a scrambled sequence. (**C**) Suppression of C/EBPβ expression, as well as levels of *Msi1, Hes1* and *Bmi1*. NTC = no-treatment control. SCR = scrambled oligonucleotide sequence RNA-transfected control. Asterisks represent significant differences (P < 0.05) compared with the NTC and SCR groups. (**D**) Western blots for Msi1, Hes1 and Bmi1 for NSCs treated with C/EBPβ siRNA. (**E**) Binding motifs for C/EBP transcription factors in the target regions used for ChIP-PCR analysis. (**F**) ChIP-PCR analysis for DNA immunoprecipitated by anti-C/EBPβ antibody.

To exert their effects, miRNAs recognize homologous sequences in the 3′UTR of target genes. However, *Msi1, Hes1* and *Bmi1* do not possess well-matched miR-155 binding sequences when examined by predictive software. Therefore, we hypothesized that regulation of *Msi1, Hes1* and *Bmi1* by miR-155 occurs indirectly via suppression of common transcription factors. By cross-referencing the consensus sequence on the promoter region of these genes with miRNA target prediction by miRanda software (http://www.microrna.org/microrna/home.do), we identified CCAAT/enhancer binding protein (C/ebp) as potential miR-155-regulated mediators of NSC self-renewal genes. In NSCs transfected with the miR-155 plasmid, only C/ebpβ was significantly suppressed among four C/ebp family members (Fig. 4A and B). Thus, we investigated the involvement of C/ebp β in regulation of *Msi1, Hes1* and *Bmi1*. Expression levels of *Msi1, Hes1* and *Bmi1* were lower in NSCs treated with *C/ebpβ* siRNA compared to the no treatment control and treatment with a scrambled oligonucleotide sequence RNA-transfected control (Fig. 4C and D). Because *Msi1, Hes1* and *Bmi1* possess consensus sequences for binding C/ebps (Fig. 4E), we performed chromatin immunoprecipitation (ChIP)-PCR assays with an anti-C/EBPβ antibody to confirm the binding of C/ebpβ. The ChIP-qPCR assays revealed that C/ebpβ binds to the promoter regions of *Msi1, Hes1* and *Bmi1* (Fig. 4F).

### *C/EBPβ, MSI1, HES1* and *BMI1* are regulated by miR-155 in human NSCs

**Figure 5:**
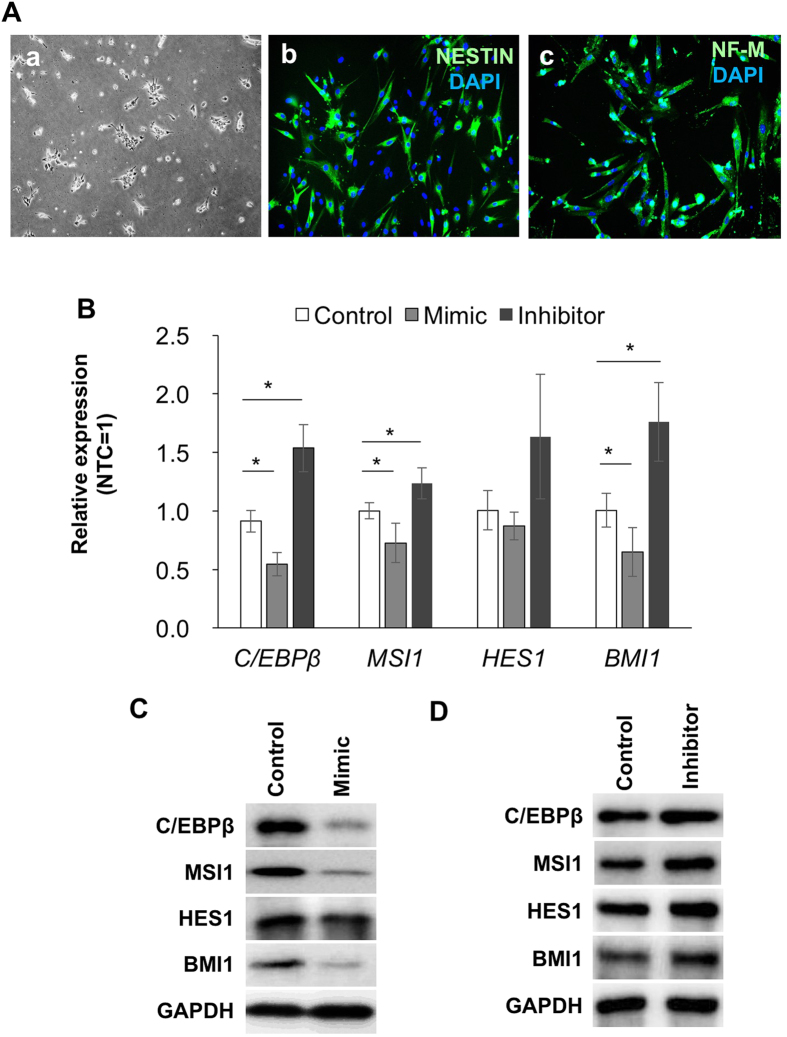
Suppression of target genes by miR-155 is conserved in human NSCs derived from human iPS cells. (**A**) NSCs induced from human iPS cells (a), and NESTIN and NF-M expressing neural cells derived from NSCs (b). (**B**) quantitative RT-PCR for *C/EBPβ, MSI1, HES1* and *BMI1* for NSCs derived from human iPS cells. The NSCs were treated with hsa-miR-155 mimic oligonucleotide (Mimic) or inhibitor to hsa-miR-155 (Inhibitor). The control was NSCs transfected with a scrambled sequence. Asterisks represent significant differences (P < 0.05) compared with the control. (**C**) Western blots for miR-155 targets for NSCs treated with the mimic oligonucleotide. (**D**) Western blots for miR-155 targets for NSCs treated with the inhibitor oligonucleotide.

Finally, we demonstrated that miR-155-induced regulation of target genes involved in self-renewal through C/EBPβ is conserved in human NSCs differentiated from human iPS cells generated using episomal plasmids. When cultured in NSC-induction medium, NSCs expanded and formed uniform colonies (Fig. 5A-a). The expanded NSCs expressed the NSC markers *SOX1, SOX2, MSI1, HES1* and *BMI1* (Data not shown). After changing the medium to neural differentiation medium, NSCs differentiated into NESTIN and NF-M-expressing neural cells (Fig. 5A-b and A-c). As observed with mouse NSCs, treatment of human iPS-derived NSCs with miR-155 mimic suppressed expression of *C/EBPβ, MSI1, HES1* and *BMI1.* Furthermore, human iPS-derived NSCs transfected with the miR155 inhibitor displayed increased expression levels of *C/EBPβ, MSI1, HES1* and *BMI1* relative to hsa-miR-155-mimic-treated cells and control cells (Fig. 5A and C).

## Discussion

NSC proliferation, differentiation and migration are controlled by a variety of intracellular and extracellular signaling events^38^,^39^. Various inflammatory cytokines are elevated in the brain following trauma, infection and neurologic/cerebrovascular disease^40^. Some of these molecules are known to induce cell death and obstruct neurogenesis^1^,^4^,^41^,^42^. Interleukin-1beta (IL-1β) is one of the most well documented cytokines activated by acute brain injury and intracerebral inflammation^40^,^41^,^43^,^44^. IL-1β inhibits neurogenesis by influencing proliferation and neural differentiation of NSCs^45^,^46^. It has also been discovered that IL-1β decreases survival of embryonic neural progenitor cells and increases differentiation toward glial lineages rather than a neuronal lineage^47^,^48^.

In this study, we examined the effect of IL-1β on mouse NSC self-renewal and found that administration of IL-1β blocked self-renewal of NSCs. We then described a mechanism for inflammation-induced inhibition of self-renewal involving miR-155. Involvement of miR-155 in inflammatory reactions has been described previously in many tissues including the brain. Woodbury *et al*. reported that miR-155 is the most highly elevated miRNA in primary microglia stimulated with lipopolysaccharide (LPS), which is a driver of acute inflammation^3^. Since miR-155 and its target genes are highly conserved across species, we hypothesized that miR-155 could be involved in stem cell maintenance and regulation of differentiation. Therefore, we examined miR-155 involvement in the IL-1β-induced suppression of stem cell self-renewal and measured expression of three central molecules involved in NSC maintenance. IL-1β significantly induced miR-155 expression 12 hours after administration, with high expression levels maintained for an additional 12 hours. Over-expression of miR-155 brought about morphological changes in the neurospheres, arrested cell proliferation and suppressed self-renewal genes, whereas treatment with miR-155-inhibitor ameliorated the IL-1β-induced effects on self-renewal by relieving suppression of *Msi1, Hes1* and *Bmi1*. These results clearly demonstrate that miR-155 inhibits NSC self-renewal, and the effects of miR-155 over-expression mimicked those observed for IL-1β treatment. The importance of the miR-155 in the stem cell regulation has been shown in the hematopoietic and immune systems. For example, sustained expression of miR-155 in mice causes polyclonal expansion of pre-leukemic pre-B cells^49^. Furthermore, granulocyte/monocyte expansion has been observed in hematopoietic stem cells in response to inflammation^50^. Moreover, mice carrying a null mutation of miR-155 display defective immune responses because of alterations in cytokine expression^51^,^52^. In addition, it has been reported that miR-155 induces irregular differentiation in neuroprogenitor cells in mice^3^. However, it remains to be elucidated how miR-155 regulates stem cell fate.

To describe the mechanism of miR-155 regulation of stem cell self-renewal, we examined the possibility that a common transcriptional factor serves as an intermediary for transcriptional regulation of *Msi1, Hes1* and *Bmi1,* since these genes lack target sequence for miR-155. The contribution of CCAAT-enhancer binding proteins C/EBPs for adult stem cell maintenance has been described previously^53^,^54^,^55^. The C/EBP family is comprised of basic leucine zipper transcription factors that participate in multiple process of tissue homeostasis through regulation of cell proliferation and differentiation^53^,^54^. C/EBPβ is thought to be particularly important for neural stem cell maintenance and neural regeneration^56^,^57^,^58^. Cortes-Cantei *et al*., showed that C/EBPβ plays a critical role in neurogenesis in the adult hippocampus, where it is expressed in the newly generated neural stem cells of the dentate gyrus (DG). Loss of C/EBPβ results in reduced cell proliferation and impaired differentiation of newly generated neurons in the adult hippocampal DG *in vivo*^57^. Zhang *et al*. demonstrated that C/EBPβ inhibits NSC differentiation^59^. To examine whether expression levels of C/EBP family members are affected by miR-155, we observed expression levels of *C/ebpα, β, γ, δ*, and *ε* in NSCs transfected with an miR-155 over-expression plasmid. We found that only C/ebpβ was suppressed by miR-155 over-expression in NSCs. Furthermore, we demonstrated that suppression of *C/ebpβ* by siRNA resulted in significant attenuation of *Msi1, Hes1* and *Bmi1* expression. Since these three genes possess CCAAT binding domains on their promoters, we performed ChIP analysis to confirm that C/ebpβ bound to the promoter regions of these genes. Until now, the idea that self-renewal genes are directly regulated by C/ebpβ in NSCs has not been documented, but these mechanisms are consistent with the previous notion that C/EBPβ plays an important role in the definition of NSC fate. On the other hand, C/EBPβ is also involved in the early differentiation stages of neural progenitors. Menard *et al*. determined that the MEK-C/EBPβ pathway is essential for differentiation of cortical progenitor cells into post-mitotic neurons^58^. Cortes-Canteli *et al*. have demonstrated that overexpression of C/EBPβ induces neuronal differentiation in a neuroblastoma cell line^60^. Furthermore, it has also been suggested that C/EBPβ works as a suppressor of gliosis and abnormal astrocyte expansion^58^,^61^. From these findings, it is clear that C/EBPβ is important for normal regeneration through differentiation activation. Interestingly, Fields *et al*. suggested that C/EBPβ expression is inducible by inflammatory molecules^62^. This finding is inconsistent with other evidence including our data that show that C/EBPβ is negatively regulated by miR-155, which is strongly induced by inflammatory molecules. It is likely that normal regeneration processes are precisely regulated by levels and timing of expression of inflammatory molecules, as well as other factors such as growth factors. However, excessive or chronic inflammations and subsequent miR-155 over-expression may disrupt the homeostasis including attenuation of C/EBPβ and its downstream genes.

Although many common neurological diseases can be modeled in mice, in many cases the results from experiments in animals are not transferrable to human cells^63^,^64^. Furthermore, miRNA sequences are not always conserved between humans and rodents. Frequently, the same miRNAs from different species do not target the same genes^65^. In this study, we confirmed our results using NSCs derived from human iPS cells to demonstrate that the molecular cascade consisting of miR-155, C/EBPβ and its targets *MSI1, HES1* and *BMI1* is conserved in human cells. Transfection of an miR-mimic for hsa-miR-155, decreased expression levels of *C/EBPβ, MSI1, HES1* and *Bmi1*, whereas these genes were upregulated by transfection of an miR-inhibitor to hsa-miR-155. Taken together, these data suggest that at least three important human genes for NSC self-renewal *MSI1, BMI1* and *HES1* are regulated by the common transcription factor C/EBPβ and that they are indirectly regulated by miR-155.

Enhancement of miR-155 has been demonstrated in various inflammation associated CNS disorders such as ALS^32^, MS^7^, Alzheimer diseases (AD) and Down’s syndrome^34^. In ALS, miR-155 is the most highly upregulated miRNA^35^. Although the detailed target genes of miR-155 in ALS remain to be elucidated, an observed increase of GFAP-positive and PSA-NCAM-positive neuroprogenitors in the subventricular zone (SVZ) suggests activation of NSCs in response to ALS^66^. Butovsky *et al*. found that peripheral injection of miR-155 inhibitor reverses abnormal molecular signature of spinal cord in the model mice, delays diseased onset and extends survival by restoring microglia dysregulation^35^. Koval *et al*. also demonstrated that treating SOD1 mutant mice with anti-miR-155 significantly extends survival reduces disease duration^32^. Also in MS, miR-155 expression is significantly increased in brain^67^, peripheral monocytes, parenchymal microglia^33^ and plasma cells^68^. In experimental autoimmune encephalomyelitis (EAE) mice, miR-155 promotes inflammation and demyelination through activation of Th17 and Th1 differentiation^68^,^69^. A direct link between NSCs and miR-155 has not been shown in MS, but close associations between inflammation and neurodegeneration in all lesions of multiple sclerosis have been suggested^70^. The inflammatory response that is the hallmark of MS not only contributes to the destruction of myelin, but may also be critical for repair and regeneration. Indeed, increased differentiation of NSCs were observed in some studies using EAE^71^,^72^. Guedes *et al*., observed upregulation of miR-155 in a transgenic Alzheimer model mice, as well as in Aβ-activated microglia and astrocytes^73^. Increased production of inflammatory mediators^73^ and activation of T-cell immune functions have been suggested as possible mechanisms^74^. Although direct evidence linking miR-155 upregulation and stem cell dysfunction have not been observed previously, production of pro-inflammatory cytokines and overexpression of the markers for early neurons such as TUC-4 and DCX have been observed in AD patients^75^. Furthermore, impaired neurogenesis in the DG and significant reduction in proliferation, survival, and migration of NSCs in the SVZ were demonstrated in transgenic mice for mutant APP and in mice infused with Aβ^76^. Based on these previous studies, it is possible that over-expressed miR-155 forces NSCs to differentiate and depletes the stem cell pool. Consistent with this notion, reduction of stem cell numbers has been observed in Down’s syndrome brains^77^. Interestingly, concomitant upregulation of miR-155 and reduction of C/EBPβ have also been observed in brains of Down’s syndrome patients^34^. Our results are consistent with these observations and reveal that a molecular axis consisting of miR-155, C/EBPβ and stem cell regulatory genes may be involved in the pathogenesis of these diseases.

Recent studies suggest that selective modulation of miRNA through antisense inhibition could be used as a therapeutic measure^25^. With advancement in the technologies for administration of miRNA, miRNA therapy becomes a real option for the management of various diseases^78^,^79^,^80^. Therapeutics using miR-155 inhibitors have also been developed for lung cancer^81^. Since a single miRNA can modify the expression of many target genes, which can work in independent molecular networks, these emerging therapeutic tools need to be carefully evaluated. However, Further studies targeting miR-155 may provide new prospects in discovering the causative factors involved in disease pathogenesis and reveal novel strategies for managing incurable neurodegenerative diseases.

## Additional Information

**How to cite this article:** Obora, K. *et al*. Inflammation-induced miRNA-155 inhibits self-renewal of neural stem cells via suppression of CCAAT/enhancer binding protein β (C/EBPβ) expression. *Sci. Rep.* **7**, 43604; doi: 10.1038/srep43604 (2017).

**Publisher's note:** Springer Nature remains neutral with regard to jurisdictional claims in published maps and institutional affiliations.

## Supplementary Material

Supplementary Information
