# Supplementary material for: Inflammation-induced miRNA-155 inhibits self-renewal of neural stem cells via suppression of CCAAT/enhancer binding protein β (C/EBPβ) expression
Source: Sci Rep. 2017 Feb 27;7:43604. doi: 10.1038/srep43604 (PMC5378916; doi:10.1038/srep43604)
Supplement: Supplementary Information [file srep43604-s1.docx]

Supplementary Table S1.

Primer sequences used for realtime RT-PCR analysis in the present study.

| Species | Gene | Forward | Reverse |
| --- | --- | --- | --- |
| Mouse | *Msi1* | GCCATGCTGATGTTCGAC | CTCTCAAACGTGACAAATCCA |
|  | *Hes1* | AAGACGGCCTCTGAGCACAG | TTATTCTTGCCCTTCGCCTC |
|  | *Bmi1* | TTTATGCAGCTCACCCGTC | CTCCTCATCTGCAACTTCTCC |
|  | *Nr2e1* | AGGAGCATTCGAAGGAATAGGAC | ACTTCCAAACACTTCTTCAGTCG |
|  | *Nestin* | ACTCTCGCTTGCAGACACC | CAAGGAAATGCAGCTTCAG |
|  | *Nf-M* | CCACGACCTCAGCAGGTAC | CATTTCCCACTTGGTTCCC |
|  | *C/ebpα* | AAGCCAAGAAGTCGGTGGAC | TCTGTTGCGTCTCCACGTTGC |
|  | *C/ebpβ* | AAGAAGACGGTGGACAAGCTG | TGCTCCACCTTCTTCTGCAGC |
|  | *C/ebpγ* | ACTACTCCAGGAGTGAATGG | TGCTTGCTTGGAGGCACAGC |
|  | *C/ebpδ* | TCCACGACTCCTGCCATGTAC | AAGAGTTCGTCGTGGCACAG |
|  | *C/ebpε* | AGACAGCCGTGCACCTCC | TGCCTTCTTGCCCTTGTGTG |
|  | *Gapdh* | TGGAGTCTACTGGTGTCTTC | TCTCGTGGTTCACACCCATC |
|  |  |  |  |
| Human | *MSI1* | TCATGCCCTACGGAATGGAC | AACTGGTAGGTGTAGCCAGG |
|  | *HES1* | AGCCAGTGTCAACACGACAC | GTGTTTTCAGCTGGCTCAGAC |
|  | *BMI1* | AGAAGGGATTTTTATGCAGC | TTCATCTGCAACCTCTCCTC |
|  | *C/EBPβ* | AGAAGGTGGAGCAGCTGTC | AGCTGCTTGAACAAGTTCC |
|  | *GAPDH* | AAGTATGACAACAGCCTCAAG | TCCACGATACCAAAGTTGTC |
